# Effects of Combining High-Definition Transcranial Direct Current Stimulation with Short-Foot Exercise on Chronic Ankle Instability: A Pilot Randomized and Double-Blinded Study

**DOI:** 10.3390/brainsci10100749

**Published:** 2020-10-17

**Authors:** Yuanbo Ma, Keyi Yin, Wei Zhuang, Cui Zhang, Yong Jiang, Jin Huang, Brad Manor, Junhong Zhou, Yu Liu

**Affiliations:** 1 School of Kinesiology, Shanghai University of Sport, Shanghai 200438, China; 1821516020@sus.edu.cn (Y.M.); yky0504@outlook.com (K.Y.); zhuangweisus@outlook.com (W.Z.); gracejoyzc@163.com (C.Z.); 1821517006@sus.edu.cn (Y.J.); 1821517005@sus.edu.cn (J.H.); 2 Shandong Institute of Sport Science, Sports Biomechanics Laboratory, Jinan 250000, China; 3 The Hinda and Arthur Marcus Institute for Aging Research, Hebrew Senior Life, Boston, MA 02131, USA; BradManor@hsl.harvard.edu; 4 Department of Medicine, Harvard Medical School, Boston, MA 02131, USA

**Keywords:** high-definition transcranial direct current stimulation, short-foot exercise, proprioception, dynamic balance function

## Abstract

(1) Background: Balance decline is highly prevalent in people suffering from chronic ankle instability (CAI). The control of balance depends upon multiple neurophysiologic systems including the activation of cortical brain regions (e.g., the primary sensorimotor cortex). The excitability of this region, however, is diminished in people with CAI. In this pilot double-blinded randomized controlled trial, we tested the effects of high-definition transcranial direct current stimulation (HD-tDCS) designed to facilitate the excitability of M1 and S1 in combination with short-foot exercise (SFE) training on proprioception and dynamic balance performance in individuals with CAI. (2) Methods: Thirty young adults completed baseline assessments including the Active Movement Extent Discrimination Apparatus (AMEDA), Joint Position Reproduction (JPR) test, Y-balance test, and the Sensory Organization Test (SOT). They were then randomized to receive a four-week intervention of SFE in combination with tDCS (i.e., HD-tDCS+SFE) or sham (i.e., control) stimulation. Baseline assessments were repeated once-weekly throughout the intervention and during a two-week follow-up period. (3) Results: Twenty-eight participants completed this study. Blinding procedures were successful and no adverse events were reported. As compared to the control group, the HD-tDCS+SFE group exhibited significant improvements in the JPR test, the Y balance test, and the SOT at different time points. No group by time interaction was observed in AMEDA test performance. (4) Conclusions: HD-tDCS combined with SFE may improve dynamic balance and proprioception in CAI. Larger, more definitive trials with extended follow-up are warranted.

## 1. Introduction

Chronic ankle instability (CAI) is highly prevalent in people who have suffered an ankle sprain [1,2] and is characterized by recurrent ankle sprains, the perception of the ankle “giving-way,” and long-term functional impairment [3]. People with CAI are also at increased risk of osteoarthritis and diminished balance when standing and walking, leading to the loss of quality of life [4,5]. It is thus of great significance to develop novel rehabilitative strategies to improve physical function in people with CAI.

Traditional rehabilitation for CAI focuses primarily on peripheral neuromuscular function of the ankle [6]. However, both spinal and supraspinal elements of the central nervous system also play an important role in the regulation of sensation and balance [7]. Ankle joint sensation and proprioception require afferent information arising from mechanoreceptors to be delivered via peripheral, spinal, and subcortical nerve pathways to sensorimotor cortical networks in the brain. CAI diminishes the quantity and quality of afferent information and results in altered activation of these cortical networks. Decreased muscle activation and increased antagonist activity [8,9] in CAI thus arises at least in part from cortical elements of the motor control system [10]. Pietrosimone et al. [11] reported that compared to those without CAI, younger adults suffering from CAI had lower excitability of the primary motor cortex (M1) as characterized by increased resting motor threshold (RMT). Strategies designed to increase the excitability of the sensorimotor cortex may thus enhance the cortical processing of afferent input related to the ankle joint, and subsequently improve balance performance in people suffering from CAI.

Transcranial direct current stimulation (tDCS) is one promising noninvasive approach that modulates the excitability of cortical regions by transferring small electrical currents via scalp electrodes. Recent studies have demonstrated that tDCS improves both static and dynamic balance in younger and older adults as well as those with neurological diseases [12,13]. Kaminski et al., for example, reported that compared to sham (i.e., control) stimulation, anodal tDCS targeting M1 enhanced balance performance in younger adults [14]. More recently, researchers have examined tDCS as an adjunct to evidenced-based interventions such as physical therapy or balance retraining. Bruce et al. [15] reported that in individuals with CAI, a 4-week intervention comprising eccentric ankle exercises and 10 sessions of anodal tDCS targeting M1, as compared to eccentric exercise combined with sham stimulation, improved cortical excitability as measured by transcranial magnetic stimulation, dynamic balance performance as measured by a hop-to-stabilization test, and muscle activation during dynamic balance test as determined by surface electromyography.

Traditional tDCS, while a promising therapeutic strategy, utilizes relatively large sponge electrodes that generate diffuse electric fields. This leads to relatively high inter-subject variance in cortical target engagement and tDCS-induced functional improvements [16]. High-definition tDCS (HD-tDCS) is a relatively new technique that uses small gel-based electrodes in combination with neuro-modeling to optimize and better control tDCS-induced electric fields [17]. In this study, we conducted a pilot randomized control trial to examine the effects of an intervention comprising short-foot exercise (SFE) combined with HD-tDCS, targeting the sensorimotor cortices on proprioception and dynamic balance in people with CAI. We hypothesized that this intervention, as compared to SFE combined with the sham (i.e., control) stimulation, would led to greater benefit to ankle-joint proprioception and dynamic balance performance in people with CAI.

## 2. Methods

### 2.1. Participants

Thirty young adults with CAI (age: 18–30 years, 15 females) were recruited. Inclusion criteria were: (1) self-reported history of the first acute ankle sprain more than one year ago; (2) experience of two or more sprains to the same ankle in the three months prior to study; (3) at least two episodes of slack or ‘giving-way’ associated with the same ankle in the six months prior to participation; (4) a Cumberland Ankle Instability Tool (CAIT) score less than 24 (questionnaire scoring ranges from 0–30, with lower score reflecting more ankle instability) [18]; and (5) the ability to stand or walk for at least 6 min. Exclusion criteria were: (1) any self-reported neurological disease other than CAI known to significantly affect motor control (e.g., cerebellar diseases, cerebral palsy, stroke, dementia, etc.); (2) history of lower extremity surgery or fracture; or (3) currently active in another rehabilitative intervention program.

Sample size estimation for this study was based upon the results from two previous studies [15,19]. One study demonstrated that 24 sessions of SFE improved joint position sense in people with CAI as compared to proprioceptive sensory exercise; that is, those who received SFE had 20 ± 14% greater improvement [19]. The other study demonstrated that 10 sessions of tDCS targeting M1 in combination with eccentric ankle exercises induced improvement in the performance of dynamic balance test in people with CAI as compared to the sham. Specifically, those who received real tDCS had 8 ± 6% greater improvement compared to those who received sham [15]. Based upon this data, we estimated that 22 participants total (11 per intervention arm) would provide at least 85% power to detect similar improvements in joint position sensation and balance test performance in the SFE+tDCS group (alpha = 0.05). We therefore recruited 30 participants to account for up to 25% attrition rate. All participants signed informed consent forms as approved by the Institutional Review Board of the Shanghai University of Sport.

### 2.2. Study Design

In this double-blinded and sham-controlled study, participants were randomized to receive 12 sessions of SFE combined with either HD-tDCS or sham (i.e., control) stimulation over a four-week period (i.e., three intervention sessions per week with a break of 24–48 h between each session). Functional assessments, consisting of proprioceptive and dynamic balance tests, were assessed at baseline, and after the first, fourth, eighth, twelfth session of intervention (i.e., marked as Week-1, Week-2, Week-3, and Week-4, respectively). The same tests were repeated at one and two weeks following the last session of intervention (i.e., marked as Week-5, Week-6).

### 2.3. Transcranial Direct Current Stimulation

**Figure 1:**
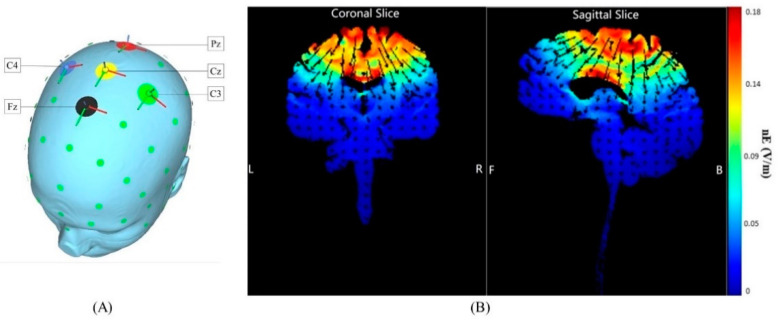
HD-tDCS electrode placement and estimated current flow within the cortex. (**A**) The anode (positive electrode) was placed over Cz of the 10/20 EEG template; the four cathodes (negative electrodes) were placed over Fz, C3, Pz, and C4. (**B**) Coronal and sagittal views of the modeled current flow depicted on a standard brain. Modeling indicated that this induced electric field influenced the foot area of the motor cortex and the primary somatosensory cortex (the area within the white circle). Warmer and cooler colors reflect the larger and smaller modeled electric field normal component (nE, V/m), respectively.

HD-tDCS was delivered by the Neuroconn system (DC-STIMULATOR, Neuroconn, Inc., Ilmenau, Germany). Finite element model analysis included in the SimNIBS software was used to optimize the tested montage (i.e., electrode placement and current parameters) [20,21]. The goal of the montage optimization was to maximize the normal component (nE) of the generated cortical electric field within the target region (i.e., the primary sensorimotor cortex (PSC)) as this component of the electric field is believed to drive modulation of neuronal excitability [22]. Five rubber round electrodes of 5 mm radius were used in the HD-tDCS montage, of which four cathodal (return) electrodes surrounded one center anodal electrode. The anode was placed at Cz of the 10/20 EEG template, and the other four electrodes were set at Fz, C3, Pz, and C4, respectively (Figure 1). Current intensity of the anode was set to 2 mA; return of current was evenly distributed between the four cathodes. Neuro-modeling results indicated that this montage induced electric fields that penetrated deep into the lower-extremity area of the primary sensorimotor cortex located along the longitudinal fissure [23,24,25].

During HD-tDCS, the current was increased from 0 mA to 2 mA over the first 30 s, maintained at this level for 19 min, and then gradually decreased to 0 mA over 30 s. For the sham stimulation, the same electrode location was used, but the current was ramped back down to 0 mA after the initial ramp-up phase. This process is commonly used as a control intervention because cutaneous sensations associated with tDCS typically fade within the first minute of stimulation [26]. After each stimulation session, participants completed a questionnaire to assess the potential side effects associated with the stimulation [27].

Double-blinding to intervention assignment was ensured by using a ‘blinded’ mode in the device software. The HD-tDCS or sham protocol was loaded into the software for each participant by study staff uninvolved in any other aspects of study. Neither the participant nor the researcher thus knew the type of stimulation being administrated.

### 2.4. Short-Foot Exercise

The goal of the SFE was to shorten the foot in the anterior–posterior direction by drawing the metatarsals to the heel without flexing the toes. This relatively novel proprioception training strategy has been shown to improve foot muscle strength and dynamic stability more than conventional proprioceptive sensory exercise [19]. Four blocks of SFE were completed during each 20 min HD-tDCS or sham stimulation session. Within each block, participants completed 12 repetitions. Approximately two minutes of rest were provided between blocks. The intensity of training was divided into three levels through which the exercise difficulty was gradually increased; that is, performance in a sitting condition, in two-leg standing condition, and in one-leg standing condition. Particularly, in the one-leg standing condition, the stability trainer provided weight-resistance to the participants. To protect participants from injury or accident events, each exercise session was carefully administrated by trained, research staff.

### 2.5. Functional Assessment

#### 2.5.1. Active Movement Extent Discrimination Apparatus (AMEDA)

AMEDA [28] was used to measure proprioception, that is, the sensitivity of the ankle joint to small differences in the degree of ankle inversion. The AMEDA instrument has a moveable footplate secured to a fixed wooden platform, where the foot is positioned along an axis that is randomly tipped to four inversion angles (Position 1 = 10°, Position 2 = 12°, Position 3 = 14°, and Position 4 = 16°), while the participant is standing and looking forward. The test requires participants to make a judgement of ankle inversion, where there were four possible responses (position 1, 2, 3, and 4) related to the four possible inversion positions (10, 12, 14, and 16 degrees). Each participant completed a familiarization session immediately before data collection. This session included moving the ankle from the smallest (position 1, i.e., 10 degrees) to largest (position 4, i.e., 16 degrees) angle and was repeated three times (i.e., 12 positions in total). Participants then completed 40 trials: 10 of each of the four different inversion displacements to the affected ankle, in a randomized order without feedback being given as to whether the judgment made for each trial was correct. The raw data were then converted into a 4 × 4 matrix. Non-parametric signal detection analysis was applied to generate receiver operating characteristic (ROC) curves, and the mean area under the ROC curve (AUC) was calculated as the score of their proprioceptive function measurement [28]. The score ranged from 0.5 to 1.0 and the higher score reflected better function.

#### 2.5.2. The Joint Position Reproduction

The joint position reproduction (JPR) test of proprioception [29] was assessed on the CON-TREX^®^ isokinetic dynamometer (Con-Trex^®^ MJ, Physiomed, Schnaittach, Germany). This test has been proven to reliably evaluate ankle joint position perception [30]. Participants were positioned supine with the leg parallel to the ground supported at the calf, the hip flexed to 45°, the knee flexed to 80°, and the talocrural joint dorsiflexed to 15°. The bare foot was aligned with the axis of the dynamometer, which was determined to be 0° and fixed on the footplate. Participants were blindfolded during the examination. Testing was performed in random order at 10° and 15° inversion, and 15° eversion. The subjects were randomly tested three times in each angle. The testing began from 0° to the preselected test position and maintained on the angle for 10 s. Thereafter, the foot passively moved back to the 0° position. Participants were then asked to move their ankle back to the test position and stop when they thought the test location was achieved. The absolute error value between the test location and the participant’s subjective joint position was used for analyses.

#### 2.5.3. Y-Balance Test

The Y-balance test, an modified version of the Star Excursion Balance Test (SEBT), was used to measure dynamic postural control [31]. This assessment measures postural control in the anterior (ANT), posteromedial (PM), and posterolateral (PL) directions. Participants placed the unstable ankle on the center block and pushed the indicator box as far as possible in the three directions with the opposite foot. Before the test, participants were instructed on how to complete the test and practiced three times in each direction. When the test officially started, participants completed three consecutive trials in each reach direction, with one minute rest in between each trial to minimize fatigue [32]. Participants were required to maintain balance while pushing the box as far as possible in the direction, and then return the foot to the center block while maintaining balance. If they lost their balance or stepped on the box during the trial, the trial was restarted [33]. In order to eliminate the influence of the length of the lower limbs, the leg length from ASIS to the medial malleolus (cm) was measured and used to normalize reach distance with the following formula [32]:

Composite reach distance = (ANT + PM + PL)/(leg length × 3) × 100(1)

#### 2.5.4. Sensory Organization Test (SOT)

The SOT was completed using a Neurocom Smart Balance Master system (Natus Medical Inc., Pleasanton, CA, USA) to quantify the relative role of proprioceptive, visual, and vestibular feedback to standing postural control [34]. The SOT was tested under six sensory conditions: eyes open on a firm surface (SOT1), eyes closed on a firm surface (SOT2), eyes open with sway referenced visual surround (SOT3), eyes open on sway referenced support surface (SOT4), eyes closed on sway referenced support surface (SOT5), eyes open with sway referenced visual surround, and sway referenced support surface (SOT6). The SOT equilibrium score provided a balance score for each condition that reflected the amount of sway in the anterior–posterior direction, where higher scores indicated better balance and less sway [35]. This score was used in the following analyses.

### 2.6. Statistical Analysis

All statistical analyses were performed using SPSS statistical software (version 21.0 for Windows; SPSS, Inc., Chicago, IL, USA). Statistical significance level was set as *p* < 0.05, 2-tailed. Two-way repeated-measures analysis of variance (ANOVA) models were used to examine the effects of SFE plus HD-tDCS on proprioception and dynamic balance function. Independent variables were group (HD-tDCS, sham) and time (baseline, Week-1, Week-2, Week-3, Week-4, Week-5, Week-6). Primary dependent variables were the AMEDA score and the Y-balance composite reach score. Secondary outcomes included the JPR absolute error value and the SOT equilibrium score. Separate models were tested for each outcome. Fisher’s least significant difference (LSD) post-hoc testing was conducted to identify differences in factor means within significance ANOVA models. Partial eta squared was used to examine the effect size of the treatment.

## 3. Results

**Figure 2:**
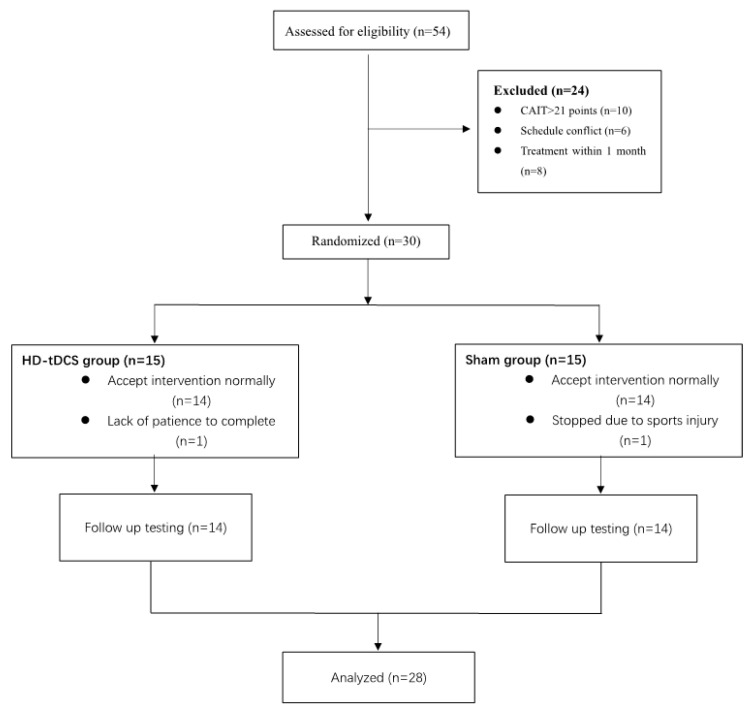
Consort patient flow chart.

Twenty-eight of 30 participants completed this study (Table 1, Figure 2). One participant withdrew due to injury deemed unrelated to study participation. Another participant withdrew due to insufficient time needed to complete the study protocol. No significant differences were observed between intervention groups in demographics or function at baseline. For blinding efficacy, nine of 14 participants who received HD-tDCS and seven of 14 participants who received the sham guessed they had received tDCS. The overall accuracy of participant guesses of intervention assignment (i.e., HD-tDCS or sham) was thus 57.1% (Fisher’s exact test; *p* = 0.833), indicating excellent blinding efficacy. No severe side effects were reported during the study (Table S1). The instrument used to test the JPR failed during Week-1 testing, but was fixed by Week-2. Week-1 data acquired from this device were thus not included in the analyses.

### 3.1. The Effects of Intervention on AMEDA

**Figure 3:**
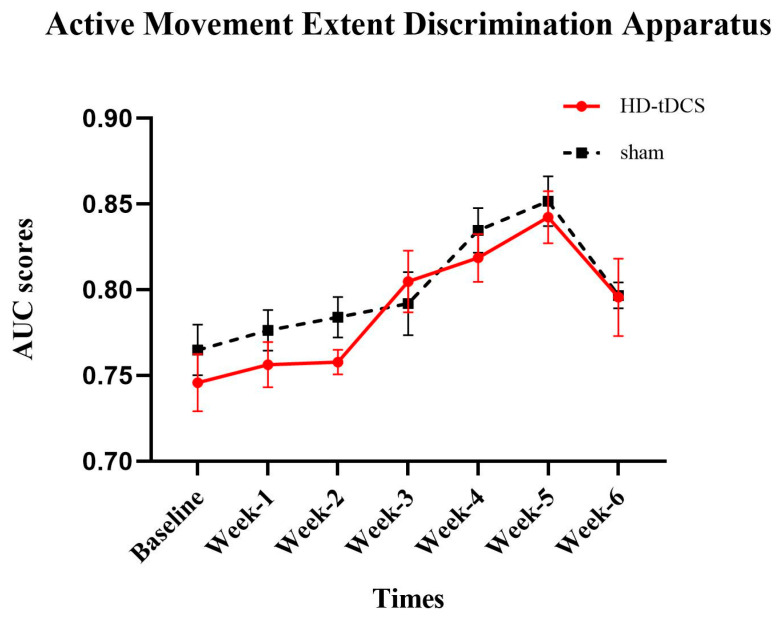
Active movement extent discrimination apparatus (AMEDA) performance (mean ± SE). Higher area under the curve (AUC) score reflects better proprioception function. Both interventions were associated with improvements compared to the baseline (*p* < 0.05).

AMEDA performance is presented in Figure 3. A significant main effect of time (F_(6156)_ = 16.240; *p* = 0.001; *η*^2^ = 0.384) was observed. Within the HD-tDCS group, post-hoc tests showed that compared to the baseline, the AMEDA score improved at Week-3 (*p* = 0.002) and that this improvement was sustained through Week-6 (*p* < 0.008). Within the sham group, compared to the baseline, an improvement appeared at Week-4 and Week-5 (*p* < 0.001).

### 3.2. The Effects of Intervention on JPR

The JPR absolute error value is presented in Table 2. ANOVA models revealed a significant time × group interaction (F_(5130)_ = 2.344; *p* = 0.029; *η*^2^ = 0.102) at 15° of inversion, while no such interaction was observed at 10° of inversion or 15° of eversion.

At 15° of inversion, a significant difference was observed between the two groups at Week-2 (*p* = 0.001). Within the HD-tDCS group, compared to the baseline, the absolute error value was lower at Week-3 and Week-6 (*p* < 0.038).

For the other two variables, the post-hoc test only showed a time effect in the 15° of eversion condition. Within the HD-tDCS group, compared to the baseline, the absolute error value was lower in Week-5 and sustained in Week-6 (*p* < 0.001).

### 3.3. The Effects of Intervention on Y-Balance Test

**Figure 4:**
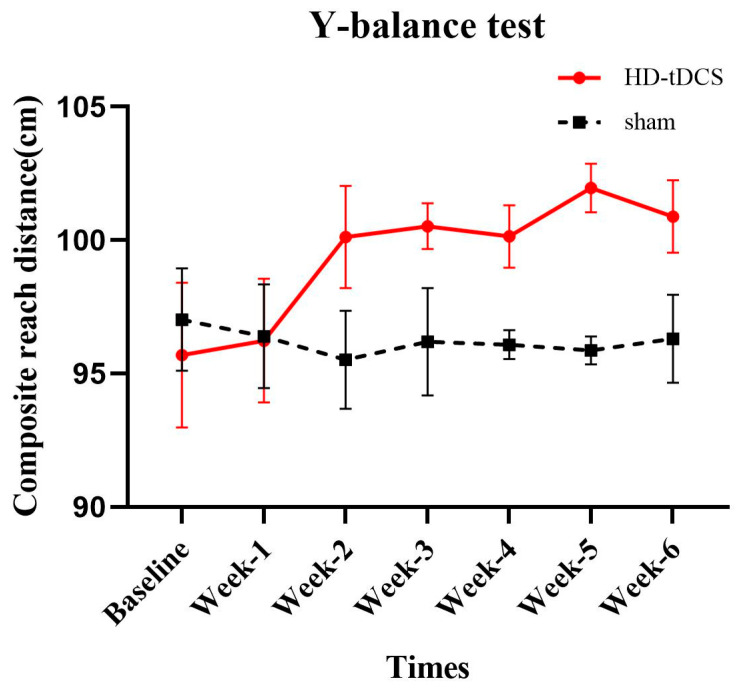
Y-balance test performance (mean ± SE). The composite reach distance in the HD-tDCS group was significantly improved (*p* < 0.05), but there was no significant change in the sham group (*p* > 0.05).

A significant time × group interaction (F_(6156)_ = 2.746; *p* = 0.044; *η*^2^ = 0.096) was observed in composite reach distance (Figure 4). Post-hoc testing revealed that a significant difference between the HD-tDCS and sham group appeared at Week-4 and that this group difference was sustained over the remaining assessments (Week-4, *p* = 0.004; Week-5, *p* < 0.001; Week-6, *p* = 0.042). Within the HD-tDCS group, compared to the baseline, the composite reach distance was significantly greater at Week-2 and such an increase was sustained through Week-6 (*p* < 0.041).

### 3.4. The Effects of Intervention on SOT

SOT equilibrium scores are presented in Table 3. A significant time × group interaction was only present for SOT4 (F_(6156)_ = 4.761; *p* = 0.002; *η*^2^ = 0.155), SOT5 (F _(6156)_ = 4.030; *p* = 0.005; *η*^2^ = 0.134), and SOT6 (F_(6156)_ = 2.586; *p* = 0.050; *η*^2^ = 0.090). A main effect of time was observed for SOT1 (F_(6156)_ = 2.895; *p* = 0.011; *η*^2^ = 0.100). No significant effects of intervention, time, or their interaction were observed for SOT2 or SOT3.

Fisher’s LSD comparisons revealed that within the HD-tDCS group, the performance of SOT4 significantly improved at Week-4 compared to the baseline and such improvement was maintained through Week-5 and Week-6 compared to the baseline. For SOT5, only the HD-tDCS group performance was greater at Week-1 compared to the baseline. This increase was sustained through Week-6. For SOT6, the performance in HD-tDCS group was significantly greater at Week-3 compared to the baseline, and this improvement was sustained through Week-6, but in the sham group, the improvement of SOT6 performance was only sustained at Week-5, and at Week-6, the improvement was eliminated.

## 4. Discussion

This randomized, sham-controlled, double-blinded pilot study indicates that SFE combined with HD-tDCS, compared to SFE combined with the sham stimulation, may result in significantly greater improvement in the performance on the Y-balance test, the SOT, and the JPR test with the ankle at 15° inversion in young adults with CAI. These results suggest that facilitating sensorimotor cortical excitability during CAI rehabilitative exercise may augment its beneficial effects on proprioception and dynamic balance performance. Larger, more definitive studies are thus warranted to confirm the results of this study.

### 4.1. Proprioception

SFE combined with HD-tDCS or the sham stimulation each similarly improved proprioception as measured by the AMEDA test. HD-tDCS thus did not augment the effects of SFE in this cohort. This may be due to the underlying pathological mechanism of CAI. Diminished proprioception in CAI arises in part from reduced mechanoreceptor sensory afferent inputs and/or weakened postural reflex responses, which is mediated by the spinal cord [29]. SFE itself enables augmentation of sensory input by increasing plantar pressures [36]. HD-tDCS, on the other hand, selectively modulates the excitability of the cerebral cortex and may thus have limited effects on the spinal components of the proprioceptive system. In a previous study, Geroin et al. [37] reported that tDCS did not enhance the effects of robot-assisted gait training on walking outcomes in patients with brain damage due to stroke, compared to robot-assisted gait training plus sham stimulation, or traditional walking training. They concluded that tDCS-induced facilitation of cortical excitability did not, therefore, influence the automaticity of walking as controlled by spinal circuits. On the other hand, in the current study, improved AMEDA performance was sustained longer in those receiving HD-tDCS (i.e., through two weeks of follow-up) compared to those receiving sham stimulation (improvements only sustained one week of follow-up). This suggests that HD-tDCS may in fact help consolidate and/or retain functional improvements, a notion that warrants future examination.

HD-tDCS combined with SFE induced significantly greater improvements in the performance of JPR at 15° of ankle inversion (but not in 15° eversion or 10° inversion), suggesting that HD-tDCS may augment SFE-induced improvement of position discrimination ability to a certain extent. This is in line with previous studies. For example, Ahn et al. [38] showed that 10 sessions of tDCS targeting C3 or C4 combined with mindfulness-based meditation in older adults with knee osteoarthritis significantly improved clinical pain and experimental pain sensitivity. Labbe et al. [39] demonstrated that tDCS targeting the right S1 improved vibrotactile detection and the discrimination threshold of the middle finger in healthy younger adults compared to sham. The diminished proprioception in CAI could lead to the overall sensory dysfunction of the ankle joint [40], which could cause dysfunction of muscles and nerves. This functional impairment may be more affected by the cerebral cortex [41]. Qi et al. [42] reported that impaired limb function could cause altered cortical excitability, and tDCS may improve damaged limb function by improving cortical processing of sensorimotor information. It should be noted that the improvement of 15° inversion in the HD-tDCS group was observed in Week-3 and Week-6, but not in Week-4. This may be due to ongoing adaptation of the participants to the change of SFE training in Week-4 from a bipedal to unipedal stance [15], a possibility supported by a similar regression observed in the sham group at this timepoint.

### 4.2. Dynamic Balance Function

We observed that the combination of HD-tDCS and SFE significantly improved the dynamic balance performance as assessed by the Y-balance test, compared to the SFE plus sham stimulation. Similar improvements were observed in the most difficult conditions of the SOT; that is, compared to the sham, HD-tDCS combined with SFE improved dynamic balance in SOT4, SOT5, and SOT6. Evidence suggests that the capacity to integrate multiple types of sensory information (e.g., visual, somatosensory and vestibular inputs) is altered in individuals suffering from CAI. When standing on unstable ground, for example, healthy people are able to maintain balance via dynamic re-weighting of sensory inputs in order to compensate for the abnormal ankle proprioceptive input [43]. Those with CAI, however, demonstrate substantially diminished postural control in this condition [44]. Moreover, patients with CAI appear to rely more heavily upon visual and vestibular information, compared to lower-extremity proprioception, to maintain balance [45]. As the processing and integration of sensory information depends upon the excitability of a distributed brain cortical network including the PSC, it is possible that tDCS-induced facilitation of PSC excitability enhanced such integration and thus improved the effectiveness of SFE [46]. At the same time, previous studies have demonstrated that, for example, tDCS targeting the temporal-parietal cortex, a region in close proximity to the PSC involved in sensory integration, can improve vestibule-perceptual sensation [47]. It is thus possible that the tDCS protocol utilized in our study also directly or indirectly modulated the excitability of these additional brain regions. Future efforts to measure brain activity during the SOT or other functional test, perhaps via EEG or near-infrared spectroscopy, both before and after intervention, are therefore needed to better understand pathways through which tDCS induces improvements in balance control.

This pilot study has several limitations. The sample size was small (n = 28) and a relatively short follow-up period was used (i.e., two weeks). As SFE was combined with HD-tDCS or sham, the effects of each intervention along with study outcomes remain unknown. Finally, as spinal and subcortical circuits undoubtedly play an important role in proprioception and its incorporation into the balance control system, future effects are needed to determine the SFE along and in compensation with HD-tDCS on the integrity of these system components. Nevertheless, this pilot study demonstrated that HD-tDCS is a promising adjunct to rehabilitation exercise programming for younger adults with CAI.

## Figures and Tables

**Table 1:** Means and standard deviations for participant demographics.

	HD-tDCS	Sham	*p*
Gender (F/M)	7/7	8/6	
Age (year)	21.14 ± 2.82	20.29 ± 1.49	0.331
Height (cm)	170.85 ± 7.35	170.96 ± 8.01	0.970
Weight (kg)	64.76 ± 13.63	62.45 ± 11.76	0.635
CAIT score	13.50 ± 4.57	15.79 ± 3.29	0.140
Affected side (L/R)	4/10	3/11	

F/M = female/male, L/R = left/right, CAIT: Cumberland Ankle Instability Tool < 24 ranging from 0 to 30.

**Table 2:** Joint position reproduction performance (mean ± SD).

Test	Group	Phase						Time × Group Effect	
		Baseline	Week-2	Week-3	Week-4	Week-5	Week-6	F	*p*
10° inversion	HD-tDCS	2.49 ± 1.0	2.66 ± 1.1	1.91 ± 0.9	2.04 ± 1.1	1.91 ± 0.5	1.88 ± 1.1	1.050	0.391
	sham	2.25 ± 0.9	2.43 ± 0.8	2.29 ± 1.3	2.51 ± 1.1	2.31 ± 1.2	1.81 ± 0.9		
15° inversion	HD-tDCS	2.66 ± 1.0	2.55 ± 0.3	1.97 ± 0.3 ^a^	2.18 ± 0.9	2.11 ± 1.1	1.77 ± 0.8 ^a^	2.951	**0.029**
	sham	2.09 ± 0.8	1.67 ± 0.5	1.91 ± 1.0	2.95 ± 1.6	1.95 ± 0.3	1.95 ± 1.3		
15° eversion	HD-tDCS	4.26 ± 1.1	3.20 ± 1.7	3.52 ± 1.9	3.19 ± 1.3	2.50 ± 1.3 ^a^	1.99 ± 0.7 ^a^	1.589	0.167
	sham	4.03 ± 1.7	3.49 ± 1.9	2.62 ± 1.1 ^a^	3.84 ± 1.4	3.07 ± 1.3 ^a^	2.50 ± 1.0 ^a^		

^a^ significant difference from baseline; *p* values ≤ 0.05 have been bolded.

**Table 3:** Sensory organization test performance (mean ± SD).

Test	Group	Phase							Time × Group Effect	
		Baseline	Week-1	Week-2	Week-3	Week-4	Week-5	Week-6	F	*p*
SOT1	HD-tDCS	94.02 ± 2.5	94.79 ± 1.6	92.91 ± 2.8	93.91 ± 2.7	93.79 ± 2.4	94.69 ± 2.1	95.39 ± 1.00 ^a^	1.220	0.299
	sham	95.19 ± 2.0	94.64 ± 2.5	93.95 ± 2.0	94.22 ± 2.1	94.26 ± 2.9	95.05 ± 1.7	94.45 ± 1.9		
SOT2	HD-tDCS	93.12 ± 3.2	93.24 ± 3.0	92.95 ± 3.1	93.41 ± 2.5	94.37 ± 2.3	93.53 ± 2.9	94.10 ± 2.0	0.994	0.431
	sham	93.81 ± 2.3	94.83 ± 1.4	94.84 ± 1.2	94.62 ± 1.7	95.11 ± 1.0	94.73 ± 1.5	94.36 ± 1.6		
SOT3	HD-tDCS	93.31 ± 2.6	94.29 ± 1.8	93.60 ± 3.1	94.26 ± 2.6	93.88 ± 2.2	93.64 ± 2.1	94.31 ± 1.9	2.081	0.058
	sham	94.47 ± 1.2	93.43 ± 2.9	94.14 ± 2.5	93.83 ± 2.9	94.29 ± 2.1	94.60 ± 1.7	93.48 ± 2.3		
SOT4	HD-tDCS	85.33 ± 5.4	83.10 ± 6.7	86.14 ± 3.5	86.74 ± 4.0	88.39 ± 2.7 ^a^	88.14 ± 3.5 ^a^	88.38 ± 2.9 ^a^	4.761	**0.002**
	sham	87.86 ± 4.0	86.34 ± 1.8	88.87 ± 1.7	87.26 ± 2.2	86.33 ± 3.8	86.48 ± 3.8	85.95 ± 4.6		
SOT5	HD-tDCS	77.08 ± 8.0	81.55 ± 5.2 ^a^	80.45 ± 3.4 ^a^	82.23 ± 2.4 ^a^	82.74 ± 3.8 ^a^	84.62 ± 4.6 ^a^	82.29 ± 3.8 ^a^	4.030	**0.005**
	sham	81.46 ± 1.1	81.01 ± 3.9	83.83 ± 2.6	82.38 ± 4.4	84.67 ± 3.5	80.24 ± 8.7	83.14 ± 5.7		
SOT6	HD-tDCS	77.24 ± 7.4	78.43 ± 2.1	80.41 ± 3.5	82.12 ± 5.0 ^a^	84.64 ± 3.0 ^a^	83.87 ± 1.2 ^a^	85.47 ± 3.1 ^a^	2.586	**0.050**
	sham	78.12 ± 6.0	81.14 ± 3.7	81.09 ± 3.7	81.61 ± 3.7	83.89 ± 1.8 ^a^	84.49 ± 1.5 ^a^	80.98 ± 7.4		

^a^ Significant difference from baseline; *p* values ≤ 0.05 have been bolded.

## Supplementary Materials

The following are available online at https://www.mdpi.com/2076-3425/10/10/749/s1, Table S1: Blinding and tolerability efficacy survey outcomes.
