# Supplementary material for: Effects of Combining High-Definition Transcranial Direct Current Stimulation with Short-Foot Exercise on Chronic Ankle Instability: A Pilot Randomized and Double-Blinded Study
Source: Brain Sci. 2020 Oct 17;10(10):749. doi: 10.3390/brainsci10100749 (PMC7602979; doi:10.3390/brainsci10100749)
Supplement: Supplementary file 1 [file brainsci-10-00749-s001.pdf]

Table S1. Blinding and tolerability efficacy survey outcomes.

Montage	Response [Real/Sham]	Confidence Level [Real/Sham]	Side Effect
Multi-target (M1+S1)	65%/35%	7.03/6.45	1.14
Sham	50%/50%	6.76/5.56	1.08

Response represents the proportion of participants who reported they believed to receive real/sham intervention. Confidence level represents the average self-reported confidence in this response, on a scale from 1 (not confident at all) to 10 (fully confident). Side effect represents self-reported side effects after the intervention, on a scale from 1 (no side effect) to 4 (serious side effect).
